# Effect of the duration of the capecitabine regimen following colon cancer surgery in an elderly population: a retrospective cohort study

**DOI:** 10.1186/s12957-021-02348-6

**Published:** 2021-08-11

**Authors:** Weiwei Chen, Hongmin Dong, Gang Wang, Juan Chen, Wenling Wang

**Affiliations:** 1 Department of Clinical Medicine, Guizhou Medical University, Guiyang, China; 2 Department of Abdominal Oncology, Guizhou Province Cancer Hospital, Guiyang, China; 3 Department of Oncology, Affiliated Hospital of Guizhou Medical University, No. 1 Beijing West Road, Guiyang, Guizhou, 550001 China

**Keywords:** Postoperative chemotherapy, Capecitabine, Colon cancer, Elderly, Survival

## Abstract

**Background:**

Only 50–70% of elderly colon cancer patients could complete the recommended 6 months of postoperative chemotherapy. It is unknown whether a shorter duration of postoperative capecitabine-alone chemotherapy would compromise survival. We thus conducted this study to analyze the association between postoperative chemotherapy duration of a capecitabine-alone regimen and cancer-specific survival (CSS) and disease-free survival (DFS) of surgery-treated elderly colon cancer patients.

**Methods:**

We performed a retrospective cohort study of surgically treated stage III and high-risk stage II colon cancer patients aged ≥ 70 treated at two medical centers. Cox proportional hazard regression models were utilized to calculate crude and adjusted hazard ratios (HRs). The nonlinear relationship between postoperative chemotherapy duration and survival was analyzed through restricted cubic spline regression analysis, and the threshold effect was calculated by the two-piecewise Cox proportional hazard model.

**Results:**

A total of 1217 surgery-treated colon cancer patients between August 1, 2013, and September 1, 2019, were reviewed, and 257 stage III and high-risk stage II patients aged ≥ 70 were enrolled. Postoperative chemotherapy with capecitabine was administered to 114 patients, and 143 patients only received surgery. As the duration of chemotherapy increased by 1 week, the risk of cancer-specific death was reduced by 11% (HR = 0.89, 95% confidence interval (CI) 0.82–0.96), and the risk of recurrence was reduced by 10% (HR = 0.90, 0.82–0.96). Nonlinearity exploration suggested a threshold effect of capecitabine duration on CSS in stage III disease. The HR for death was 0.79 (95% CI, 0.68–0.92) with duration ≤ 16 weeks and 1.34 (95% CI, 0.91–1.97) with duration > 16 weeks.

**Conclusions:**

The postoperative capecitabine duration was significantly associated with a decrease in death risk and recurrence risk in elderly colon cancer patients. However, the threshold effect of capecitabine duration on survival suggests that short-term chemotherapy may improve survival in elderly stage III colon cancer patients.

## Introduction

For several decades, a 6-month chemotherapy regimen has been the standard postoperative treatment of colon cancer [1, 2]. However, recent studies from the International Duration Evaluation of Adjuvant Therapy collaboration (IDEA) suggested noninferiority of a shorter duration (3 months) of chemotherapy, as compared with 6 months, which was confirmed in most colon cancer patients receiving the CAPOX (oxaliplatin plus capecitabine) regimen, but not in those with the FOLFOX (oxaliplatin plus 5-fluorouracil/leucovorin) regimen [3, 4]. The duration effect appeared to be dependent on the chemotherapy regimen, and it seems that the use of capecitabine could shorten the postoperative chemotherapy duration without compromising survival.

Although previous retrospective studies have revealed the benefit of adjuvant chemotherapy in elderly patients, the appropriate duration of treatment for these patients is unclear [5, 6]. To date, only a small fraction of the fittest elderly individuals have been enrolled in randomized controlled trials (RCTs). In addition, the participants in the current RCTs all received an oxaliplatin-containing regimen, but it is still controversial to administer oxaliplatin-containing regimens for patients 70 years or older. A fluoropyrimidine alone regimen is still the recommended postoperative protocol for elderly patients in clinical practice [5, 7]. The limited number of elderly participants and the different recommended chemotherapy regimens for the elderly make it impossible to evaluate the effect of capecitabine duration on survival in the elderly based on the results of RCTs. Additionally, more than 30–50% of elderly patients could not complete an entire 6-month regimen [8, 9]. We thus conducted this study to explore the association between the postoperative chemotherapy duration of the capecitabine-alone regimen and cancer-specific survival (CSS) of elderly colon cancer patients.

## Materials and methods

### Study population

**Fig. 1:**
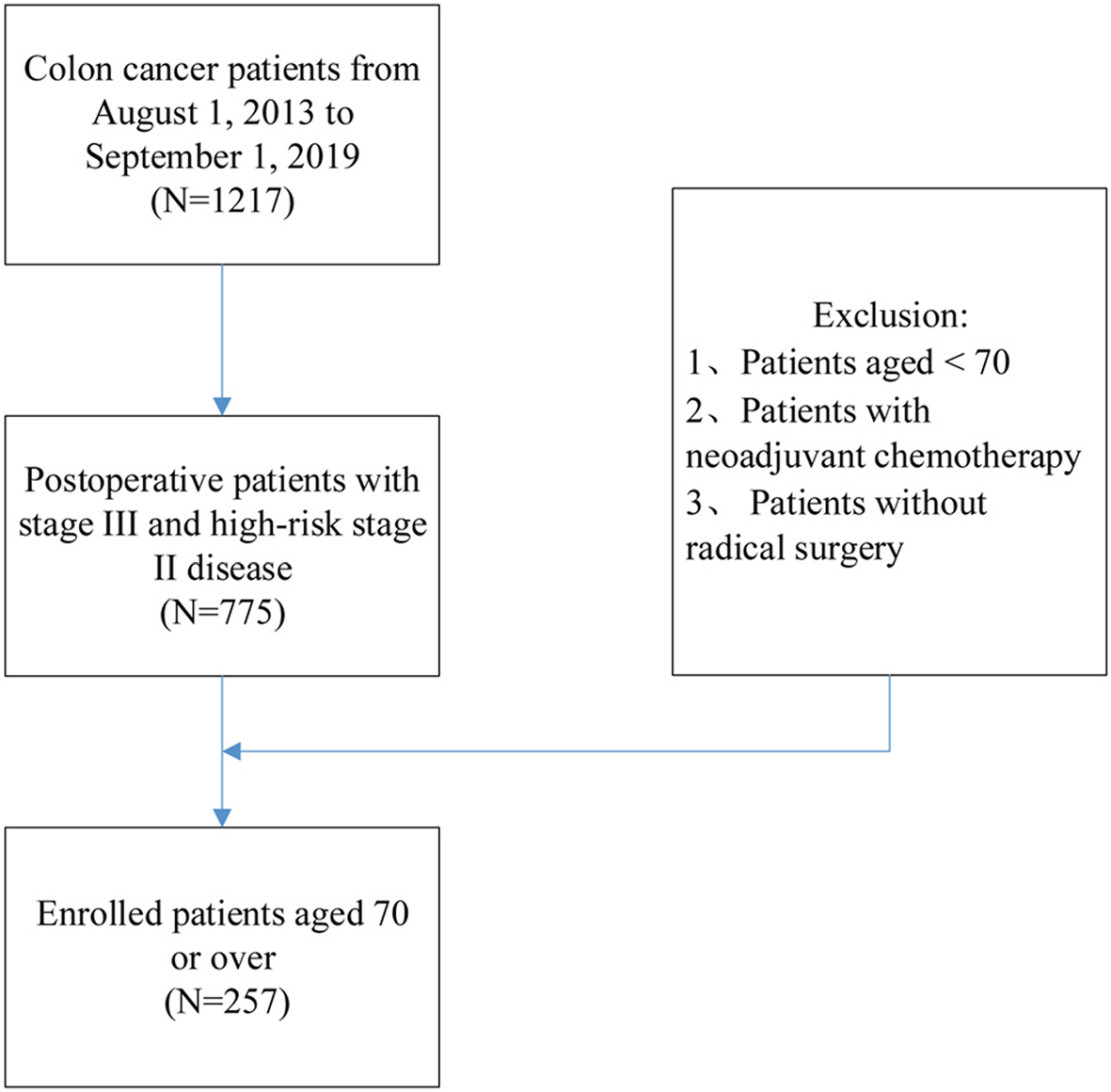
Flow chart of enrollment

Patient data were collected from the electronic medical record system of Guizhou Province Cancer Hospital and the Affiliated Hospital of Guizhou Medical University. We included high-risk stage II and stage III patients who were aged ≥ 70 years and underwent complete mesocolic excision with lymph node dissection (Fig. 1). After surgery, the patients were recommended to receive capecitabine adjuvant chemotherapy (1000 mg/m^2^ twice daily for 14 days followed by a 7-day rest period). The local ethics committee of Guizhou Province Cancer Hospital approved this study (2020-FZ 00,513).

### Study design and variables

This retrospective cohort study aimed to assess the association between postoperative capecitabine duration and CSS and DFS in elderly colon cancer patients. From August 2013 to September 2019, a total of 1217 surgery-treated colon cancer participants were retrospectively reviewed. The variables used to establish the multivariate adjusted models included (1) continuous variables: age, carcinoma antigen 199 (CA199, obtained before surgery), carcinoembryonic antigen (CEA, obtained before surgery), CCI (calculated using data regarding complications) [10], and (2) categorical variables: sex, T stage, N stage, histological type, and MMR status.

### Follow-up procedure

Follow-up for enrolled patients through outpatient visits was applied once every 3 months for the first 2 years, every 6 months for the next 3 years, and every 1 year thereafter. A complete physical examination, thoraco-abdominal CT, pelvic CT, serum CEA and CA199 assessment, and an annual colonoscopy were conducted at each follow-up. The last date of follow-up was September 1, 2020. The follow-up information was obtained by the second and third authors from the electronic medical record system of Guizhou Province Cancer Hospital and the Affiliated Hospital of Guizhou Medical University.

### Statistical analysis

Continuous variables are described as the mean ± standard, and categorical variables are described as frequency or percentage. Student’s *t* test (normal distribution), Mann–Whitney *U* test (skewed distribution), or *χ*^2^ test (categorical variables) was adopted as appropriate to investigate differences in the characteristics of the enrolled patients among the various postoperative chemotherapy duration groups. A univariate Cox proportional hazard model and a multivariate Cox proportional hazard model were applied to assess variables that may correlate with the risk of death. Three models were constructed: model 1, not adjusted for any covariate; model 2, adjusted for gender and age; and model 3, adjusted for the covariates presented in Table 1. Stratified analyses were performed by Cox proportional hazard models. A Cox proportional hazards regression model with cubic spline functions and smooth curve fitting was applied to explore the nonlinear relationship between postoperative chemotherapy duration and CSS. If nonlinearity was detected, then the inflection point was calculated by the recursive algorithm, and a two-piecewise Cox proportional hazard model was established on both sides of the inflection point. Finally, which model was more suitable for fitting the association between the target-independent variable and the outcome variable was determined by the log-likelihood ratio test.

**Table 1:** Characteristics of enrolled patients

Characteristics	Postoperative capecitabine duration			*P* value
	0	≤ 12 weeks	≤ 24 weeks	
No. of participants	143	79	35	
Age (years), mean (SD)	77.69 (4.90)	73.57 (2.77)	73.34 (2.99)	< 0.001
≥ 70, < 75	43 (30.07%)	46 (58.23%)	25 (71.43%)	
≥ 75, < 80	43 (30.07%)	33 (41.77%)	8 (22.86%)	
≥ 80	57 (39.86%)	0 (0.00%)	2 (5.71%)	
Gender, *N* (%)				0.421
Male	85 (59.44%)	54 (68.35%)	22 (62.86%)	
Female	58 (40.56%)	25 (31.65%)	13 (37.14%)	
T stage, *N* (%)				0.177
1–2	8 (5.59%)	2 (2.53%)	1 (2.86%)	
3	118 (82.52%)	68 (86.08%)	25 (71.43%)	
4	17 (11.89%)	9 (11.39%)	9 (25.71%)	
N stage, *N* (%)				0.832
0	74 (51.75%)	36 (45.57%)	18 (51.43%)	
1	57 (39.86%)	33 (41.77%)	14 (40.00%)	
2	12 (8.39%)	10 (12.66%)	3 (8.57%)	
Stage, *N* (%)				0.664
II	74 (51.75%)	36 (45.57%)	18 (51.43%)	
III	69 (48.25%)	43 (54.43%)	17 (48.57%)	
Histological type				0.883
Common type	122 (85.31%)	68 (86.08%)	31 (88.57%)	
Special type^a^	21 (14.69%)	11 (13.92%)	4 (11.43%)	
CA199, *N* (%)				0.232
Normal	122 (85.31%)	60 (76.92%)	27 (77.14%)	
Elevated	21 (14.69%)	18 (23.08%)	8 (22.86%)	
CEA, *N* (%)				0.830
Normal	92 (64.34%)	48 (60.76%)	23 (65.71%)	
Elevated	51 (35.66%)	31 (39.24%)	12 (34.29%)	
MMR status				0.021
dMMR	15 (10.49%)	3 (3.80%)	2 (5.71%)	
pMMR	106 (74.13%)	51 (64.56%)	28 (80.00%)	
Unknown	22 (15.38%)	25 (31.65%)	5 (14.29%)	
Charlson comorbidity index				< 0.001
≤ 4	88 (61.54%)	75 (94.94%)	34 (97.14%)	
> 4	55 (38.46%)	4 (5.06%)	1 (2.86%)	

Notes: ^a^Special type adenocarcinoma includes mucinous, signet ring cell, medullary, and undifferentiated variants

Abbreviations: *CA* carcinoma antigen; *CEA* carcinoembryonic antigen; *MMR* mismatch repair; *dMMR* mismatch repair deficiency; *pMMR* proficient mismatch repair

We conducted a series of sensitivity analyses to guarantee the robustness of the data analysis. First, due to the limitations of the Cox proportional hazards model in addressing nonlinearity, a generalized additive model was used to adjust the covariates; we then compared its effect size with the fully adjusted model. Second, we used different covariates as the stratification variables to observe the trend of HR among the different subgroups and calculated the *P* for trend. All data analysis was performed with the statistical software packages R (http://www.R-project.org, The R Foundation) and EmpowerStats (http://www.empowerstats.com, X&Y Solutions, Inc., Boston, MA, USA). *P* values < 0.05 (two-sided) were considered statistically significant.

## Results

### Baseline characteristics of the enrolled patients

From August 2013 to September 2019, a total of 1217 surgery-treated colon cancer patients from Guizhou Province Cancer Hospital or the Affiliated Hospital of Guizhou Medical University were retrospectively reviewed, and 257 elderly patients with stage III and high-risk stage II disease were enrolled in the final analysis. Postoperative chemotherapy with a capecitabine-alone regimen was administered to 114 (114/257, 44.36%) patients, and 143 patients only received surgery. A total of 43 patients died of colon cancer (43/257, 16.73%) during follow-up, and 8 (8/257, 3.11%) were lost to follow-up. The median follow-up time was 30.4 months. Table 1 describes the baseline characteristics of the enrolled patients across the categories of chemotherapy duration. No statistically significant difference was found in the measures except for age, MMR status, and CCI among the different chemotherapy duration groups. Compared to patients receiving postoperative chemotherapy ≤ 12 weeks or > 12 weeks, those who did not receive chemotherapy were older. Moreover, patients without postoperative chemotherapy had higher rates of CCI and MMR deficiency (dMMR) incidents.

### Association between postoperative chemotherapy duration and survival

**Fig. 2:**
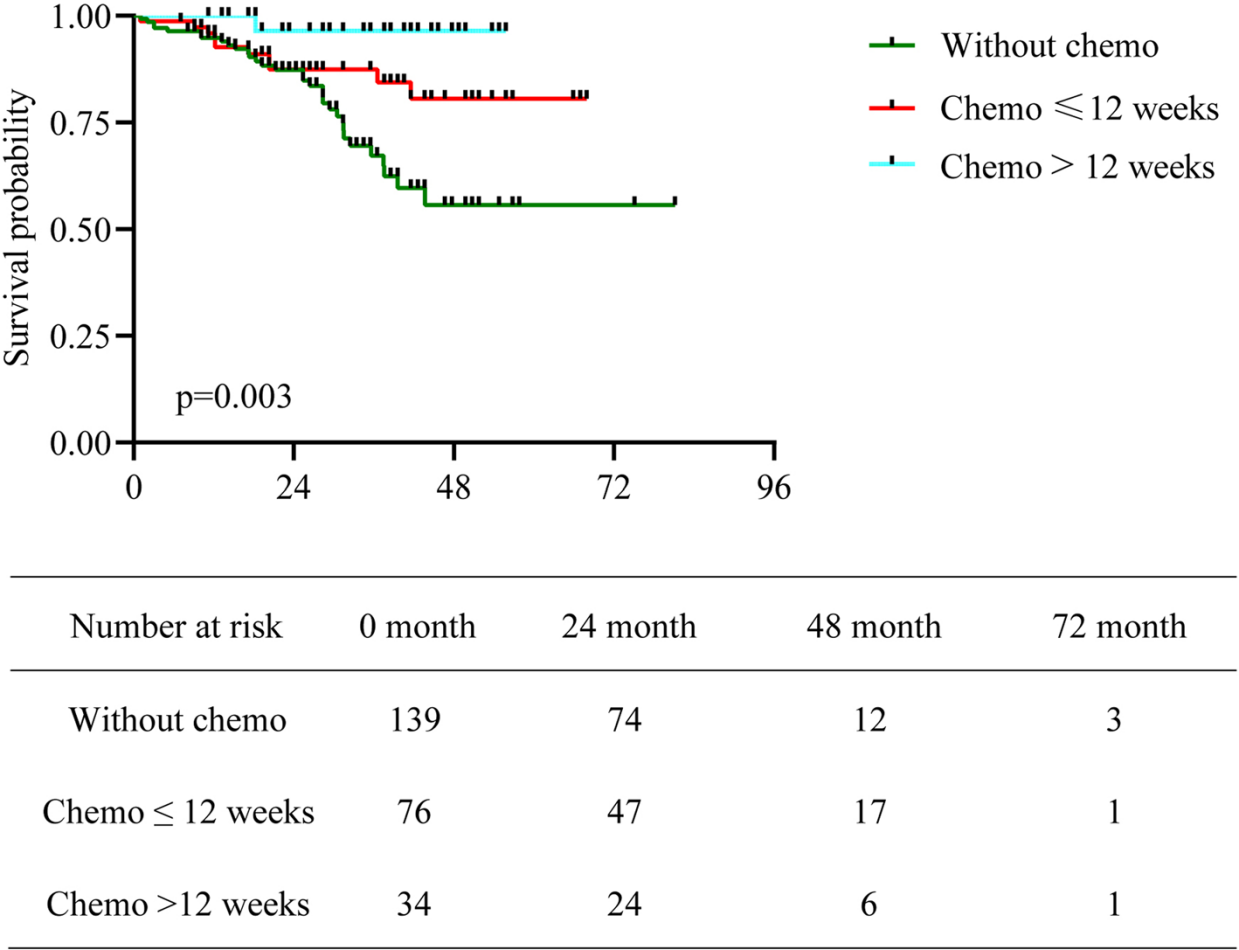
Kaplan–Meier curves of CSS in elderly patients with high-risk stage II or stage III disease. Differences among the three groups were significant (*P* = 0.003)

We evaluated the median CSS by the Kaplan–Meier method with a log-rank test. As shown in Fig. 2, the median CSS was 25.4 months (95% confidence interval (CI), 1.0–81.1) for the group without adjuvant chemotherapy, 27.4 months (95% CI, 1.0–67.9) for the group with chemotherapy ≤ 12 weeks and 35.5 months (95% CI, 11.2–84.2) for the group with chemotherapy > 12 weeks. The differences among the groups were significant (*P* = 0.003).

The results of the univariate analysis are shown in Table 2. Patients with postoperative capecitabine ≤ 12 weeks or > 12 weeks were associated with a better DFS and CSS than those treated with surgery alone. N2, stage III, and elevated CA199 were associated with poor DFS and CSS.

**Table 2:** Univariate analysis of enrolled patients

Variables	Mean ± SD/N (%)	Disease-free survival	Cancer-specific survival
		HR (95% CI), *P* value	HR (95% CI), *P* value
Postoperative capecitabine duration	4.65 ± 6.75	0.91 (0.86, 0.98), 0.014	0.90 (0.84, 0.97), 0.0043
0	143 (55.64%)	1.0	1.0
≤ 12 weeks	79 (30.74%)	0.52 (0.36, 0.98), 0.030	0.48 (0.24, 0.99), 0.046
≤ 24 weeks	35 (13.62%)	0.26 (0.19, 0.70), 0.007	0.09 (0.01, 0.67), 0.018
Gender			
Male	161 (62.65%)	1.0	1.0
Female	96 (37.35%)	1.07 (0.58–1.68), 0.21	1.12 (0.60, 2.08), 0.73
Age (years)	75.83 ± 4.60	1.04 (0.73–1.44), 0.77	1.06 (0.99, 1.12), 0.080
≥ 70, < 75	114 (44.36%)	1.0	1.0
≥ 75, < 80	84 (32.68%)	1.23 (0.73–2.88), 0.079	1.57 (0.77, 3.19), 0.21
≥ 80	59 (22.96%)	1.42 (0.75–3.13), 0.23	1.79 (0.83, 3.87), 0.14
T stage			
1–2	11 (4.28%)	1.0	1.0
3	211 (82.10%)	1.40 (0.65–5.35), 0.42	1.30 (0.31, 5.44), 0.71
4	35 (13.62%)	1.22 (0.92–5.57), 0.069	1.13 (0.23, 5.62), 0.88
N stage			
0	128 (49.81%)	1.0	1.0
1	104 (40.47%)	1.10 (0.67–3.64), 0.45	1.77 (0.85, 3.67), 0.13
2	25 (9.73%)	4.04 (1.93–8.99), < 0.0001	5.31 (2.42, 11.64), < 0.0001
Stage			
II	128 (49.81%)	1.0	1.0
III	129 (50.19%)	2.19 (1.13, 4.66), 0.017	2.45 (1.26, 4.78), 0.0083
Histological type			
Common type	221 (85.99%)	1.0	1.0
Special type	36 (14.01%)	1.28 (0.75–2.96), 0.061	1.69 (0.83, 3.44), 0.15
CEA			
Normal	163 (63.42%)	1.0	1.0
Elevated	94 (36.58%)	1.29 (0.88–2.42), 0.38	1.15 (0.62, 2.12), 0.66
CA199			
Normal	209 (81.64%)	1.0	1.0
Elevated	47 (18.36%)	2.68 (1.98–3.93), 0.0075	2.04 (1.04, 3.98), 0.037
Charlson comorbidity index			
≤ 4	197 (76.65%)	1.0	1.0
> 4	60 (23.35%)	1.18 (0.61–3.09), 0.87	1.05 (0.50, 2.19), 0.90
MMR status			
dMMR	20 (7.78%)	1.0	1.0
pMMR	185 (71.98%)	0.79 (0.42, 1.93), 0.57	0.51 (0.21, 1.23), 0.1338
Unknown	52 (20.23%)	0.67 (0.29, 1.84), 0.12	0.63 (0.22, 1.76), 0.3769

Abbreviations: *HR* hazard ratio; *CI* confidence interval; *CA* carcinoma antigen; *CEA* carcinoembryonic antigen; *MMR* mismatch repair; *dMMR* mismatch repair deficiency; *pMMR* proficient mismatch repair

As reported in Tables 3 and 4, three models were used to analyze the independent association of postoperative capecitabine duration on CSS and DFS. In the unadjusted model shown in Table 3, 0.90 (HR) demonstrated that the risk of cancer-specific death was reduced by 10% as the capecitabine duration increased by 1 week. In the adjusted model 1, after adjusting for sex and age, the negative relationship was still robust. In the adjusted model 2, we adjusted for sex, age, stage, CEA (elevated, normal), CA199 (elevated, normal), CCI (≤ 4, > 4), histological type, and MMR status as confounding factors. After adjusting for confounding factors, every additional week of capecitabine duration was associated with an 11% decrease in the risk of death. As shown in Table 4, each additional week of capecitabine correlated with a 10% decrease in the risk of recurrence. Then, postoperative capecitabine duration was transformed from a continuous variable to a categorical variable for the sensitivity analysis. The results of postoperative capecitabine duration as a categorical variable were consistent with the results when the capecitabine duration was a continuous variable.

**Table 3:** Results of multivariable analysis for CSS

	Non-adjusted^a^	Adjust I^b^	Adjust II^c^
	HR (95% CI), *P* value	HR (95% CI), *P* value	HR (95% CI), *P* value
Postoperative capecitabine duration	0.90 (0.84, 0.97), 0.0043	0.90 (0.84, 0.97), 0.0076	0.89 (0.82, 0.96), 0.0035
0	1.0	1.0	1.0
≤ 12 weeks	0.48 (0.24, 0.99), 0.046	0.46 (0.21, 0.98), 0.045	0.37 (0.17, 0.82), 0.015
≤ 24 weeks	0.09 (0.01, 0.67), 0.018	0.09 (0.01, 0.69), 0.020	0.07 (0.01, 0.56), 0.012

Notes: ^a^Non-adjusted model adjusted for: none

^b^Adjust I adjust for: gender; age

^c^Adjust II adjust for: gender; age; stage; CEA; CA199; CCI; histological type, and MMR status

Abbreviations: *HR* hazard ratio; *CI* confidence interval; *CA* carcinoma antigen; *CEA* carcinoembryonic antigen; *MMR* mismatch repair; *CCI* Charlson comorbidity index

**Table 4:** Results of multivariable analysis for DFS

	Non-adjusted^a^	Adjust I^b^	Adjust II^c^
	HR (95% CI), *P* value	HR (95% CI), *P* value	HR (95% CI), *P* value
Postoperative capecitabine duration	0.91 (0.86, 0.98), 0.014	0.90 (0.83, 0.98), 0.012	0.90 (0.82, 0.96), 0.006
0	1.0	1.0	1.0
≤ 12 weeks	0.52 (0.36, 0.98), 0.030	0.52 (0.35, 0.98), 0.033	0.45 (0.32, 0.90), 0.030
≤ 24 weeks	0.26 (0.19, 0.70), 0.007	0.26 (0.19, 0.70), 0.008	0.24 (0.17, 0.68), 0.005

Notes: ^a^Non-adjusted model adjusted for: none

^b^Adjust I adjust for: gender; age

^c^Adjust II adjust for: gender; age; stage; CEA; CA199; CCI; histological type, and MMR status

Abbreviations: *HR* hazard ratio; *CI* confidence interval; *CA* carcinoma antigen; *CEA* carcinoembryonic antigen; *MMR* mismatch repair; *CCI* Charlson comorbidity index

### Sensitivity analysis

**Fig. 3:**
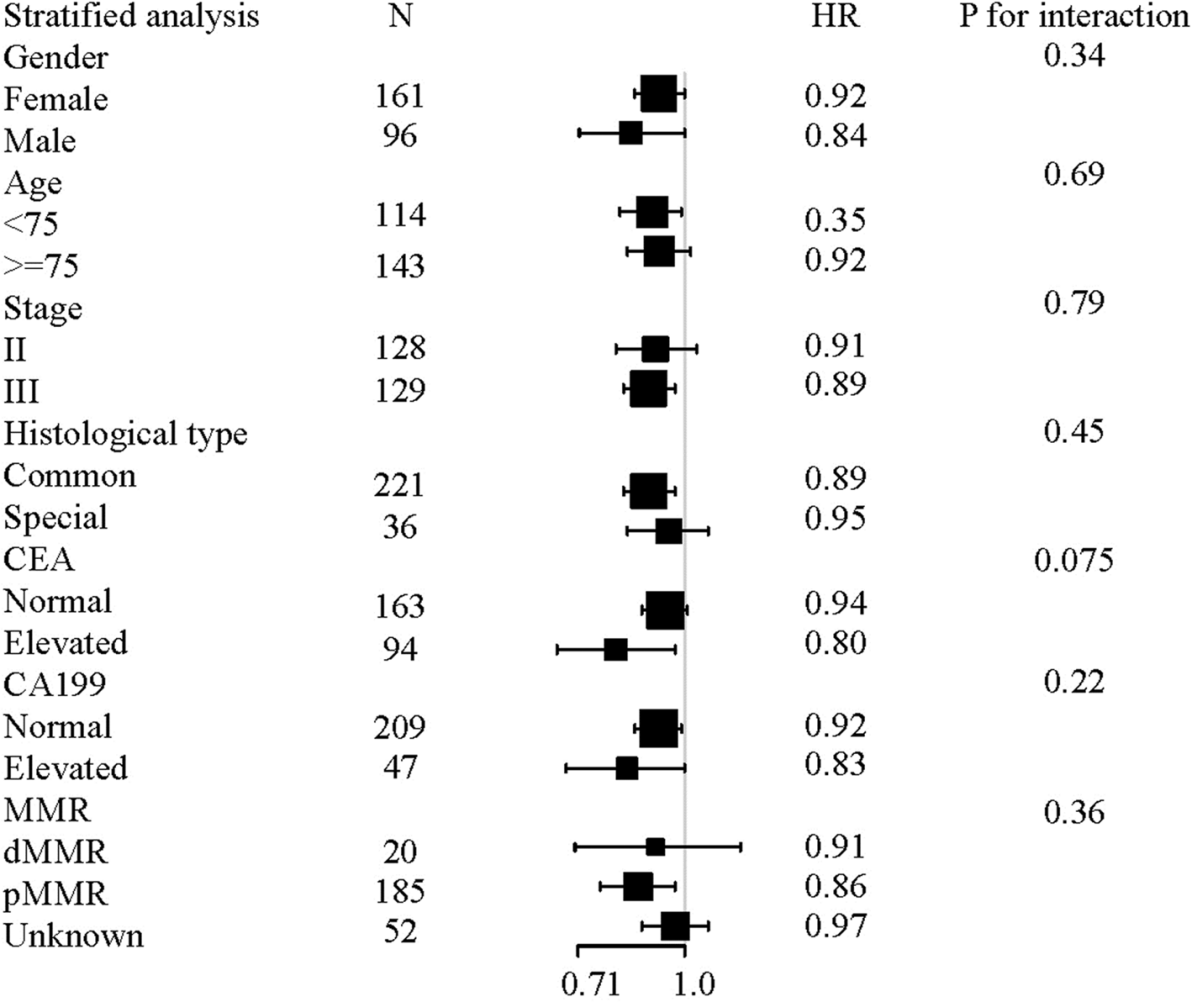
Subgroup analyses by sex, age, stage, histological type, CEA, CA199, and MMR status in stage III and high-risk stage II colon cancer

For the sensitivity analysis, we first set up model 2, which adjusted for sex, age, stage, CEA (elevated, normal), CA199 (elevated, normal), CCI (≤ 4, > 4), histological type, and MMR status. The results were consistent with those of the crude model and model 1. We then used different covariates as the stratification variables to observe the trend of HR among the different subgroups (Fig. 3). The core results were consistent with our initial cohort. The tests for interactions were not statistically significant for age, sex, stage, histological type, CEA, CA199, or MMR status (*P* > 0.05).

### Nonlinear relationship of postoperative chemotherapy duration with CSS in stage III patients

**Fig. 4:**
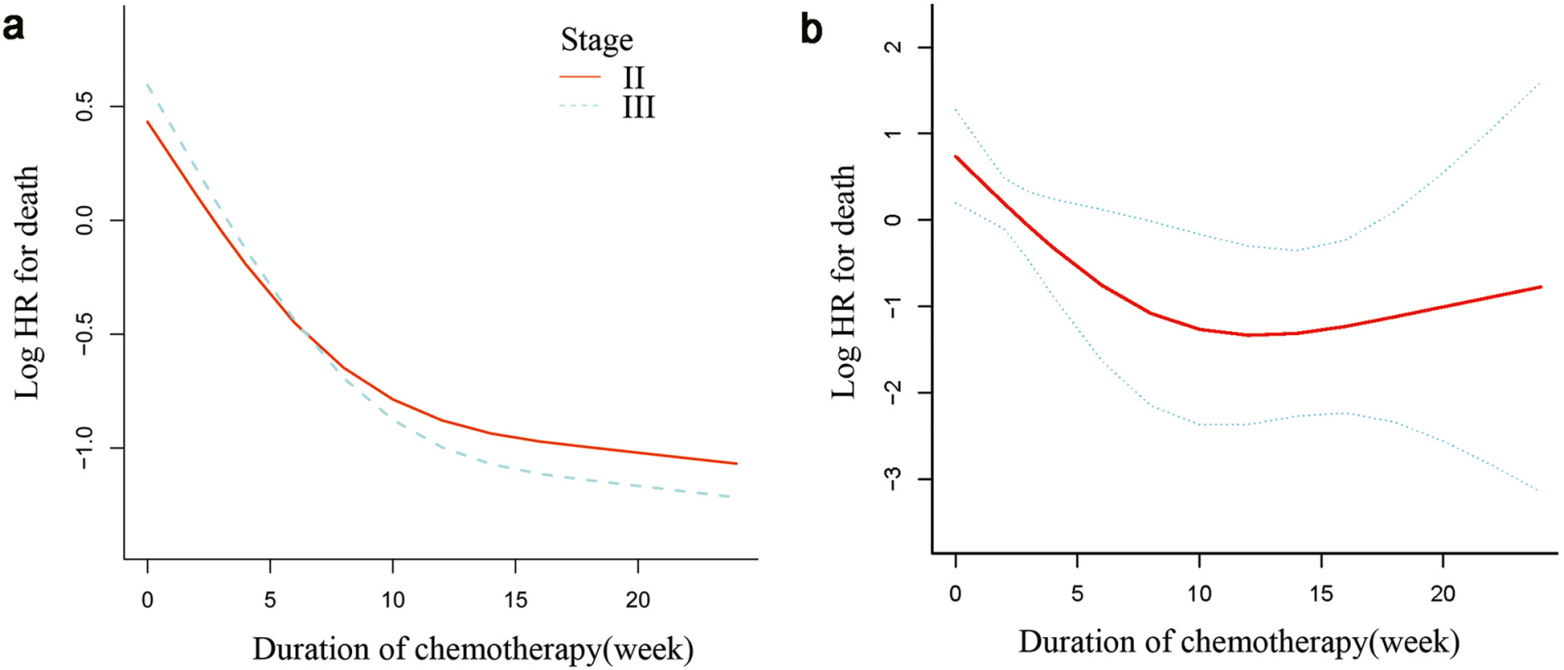
(**a**) A nonlinear relationship between postoperative capecitabine duration and CSS was observed in stage III disease (*P* for nonlinearity = 0.003) but not in stage II disease (*P* for nonlinearity = 0.24). (**b**) The red curved line shows an L-shaped relationship between capecitabine duration and the risk of death in stage III elderly colon cancer after adjusting for sex, age, T stage, CA199, CEA, histological type, CCI, and MMR status. The area between the two blue dotted lines (upper limit and lower limit) is expressed as a 95% CI. The HR for death was 0.79 (95% CI, 0.68–0.92) with a postoperative capecitabine duration ≤ 16 weeks and 1.34 (95% CI, 0.91–1.97) with a duration > 16 weeks

In our study, the nonlinearity of postoperative capecitabine duration on CSS was observed in stage III (Fig. 4a, P for nonlinearity = 0.003) but not in high-risk stage II (*P* for nonlinearity = 0.24). After adjusting for sex, age, T stage, CA199, CEA, histological type, and CCI and MMR status, the smooth curve analysis and the Cox proportional hazards regression model with cubic spline functions indicated that the relationship between the postoperative capecitabine duration and CSS was still nonlinear and presented an L-shape in stage III elderly patients (Fig. 4b, P for nonlinearity = 0.01). Both the Cox proportional hazard model and the two-piecewise Cox proportional hazard model were applied to fit the association. We selected the best fit model according to *P* of the log-likelihood ratio test.

Because its *P* for the log-likelihood ratio test was < 0.05, we selected the two-piecewise Cox proportional hazard model for fitting the association between postoperative capecitabine duration and CSS in stage III elderly patients. The inflection point was calculated as 16 by using the recursive algorithm and two-piecewise Cox proportional hazard model (Table 5). The HR for death was 0.79 with a postoperative capecitabine duration ≤ 16 weeks and 1.34 with a duration > 16 weeks. However, in high-risk stage II patients, we did not observe this nonlinear association.

**Table 5:** Results of the two-piecewise linear regression model in stage III elderly patients for CSS

	HR (95% CI), *P* value^a^
Postoperative capecitabine duration	0.86 (0.78, 0.96), 0.0046
Inflection point of duration	
< 16	0.79 (0.68, 0.92), 0.0027
≥ 16	1.34 (0.91, 1.97), 0.13
*P* for log likelihood ratio test	0.044

Notes: ^a^Adjust for: gender, age, T stage, CA199, CEA, histological type, CCI, and MMR

Abbreviations: *HR* hazard ratio; *CI* confidence interval; *CA* carcinoma antigen; *CEA* carcinoembryonic antigen; *MMR* mismatch repair; *CCI* Charlson comorbidity index

## Discussion

In this retrospective cohort study, we investigated the association of the postoperative chemotherapy duration of the capecitabine-alone regimen with CSS and DFS of surgery-treated elderly colon cancer patients. In general, as the duration of treatment increased by 1 week, the risk of cancer-specific death was reduced by 11% (HR = 0.89, 0.82–0.96), and the risk of recurrence was reduced by 10% (HR = 0.90, 0.82–0.96), which was consistent with findings mainly obtained from younger patients [11, 12]. Further analyses in the stage III group unveiled some novel findings. Nonlinearity exploration suggested an L-shaped relationship between postoperative capecitabine duration and CSS. After adjusting for potential confounding factors, the nonlinear relationship between capecitabine duration and CSS was still significant (Fig. 4b, Table 5). The HR for death was 0.79 (95% CI, 0.68–0.92) with chemotherapy duration ≤ 16 (weeks) and 1.34 (95% CI, 0.91–1.97) for a duration > 16 (weeks).

The duration effect appeared to be dependent on the use of capecitabine since patients with pT4 or pN2 tumors are still being recommended to undergo a 6-month FOLFOX regimen, while the CAPOX duration could be shortened to 3 months regardless of high-risk factors [11]. Although the capecitabine-alone regimen remains one of the recommended postoperative protocols for the elderly, some studies have reported that patients over age 65 years benefitted from the addition of oxaliplatin for trials conducted 2004–2009 but not trials conducted 1998–2003 [7]. The change may be derived from advances in supportive care. Nevertheless, undernourishment and nutritional risk in Chinese elderly patients, along with insufficient supportive therapy in China, may hinder elderly patients from benefitting from two-agent chemotherapy [13]. Even with single-agent chemotherapy, less than 50–70% of elderly patients could complete a 6-month regimen.

Our study found that in stage III elderly colon cancer patients, the effect of capecitabine duration on CSS changed at the turning point of 16 weeks. That is, after 16 weeks of capecitabine use, the declining trend of death risk as chemotherapy duration increased disappeared. To the best of our knowledge, this study is the first to demonstrate a threshold effect of postoperative capecitabine duration on CSS in elderly colon cancer patients. A 16-week duration of capecitabine chemotherapy may be an alternative to the conventional 6-month postoperative chemotherapy regimen for elderly patients with stage III colon cancer.

A threshold effect of capecitabine duration on survival, which we discovered in the stage III group, was not detected in the stage II group. The prognosis of stage II colon cancer varies significantly according to different risk factors [14–16]. The 5-year survival rate of patients with stage IIB/IIC (T4) colon cancer is significantly lower than that of patients with stage IIIA [17]. In addition, the prognosis of stage II patients with residual tumor is worse than that of stage III patients, and the incidence of postoperative tumor residual in the IIB/IIC stage is significantly higher than that in stage IIIA [18]. Therefore, patients with certain high-risk factors in stage II may need a longer capecitabine duration. A recent study also found that biomarkers could identify patients at very high risk of recurrence of colon cancer, such as CDX2 expression, which could be taken into consideration for decision-making [19]. A multicenter large cohort study may be needed to identify the effect of postoperative capecitabine duration on survival in terms of different high-risk factors in stage II.

Although the International Society of Geriatric Oncology (SIOG) has recommended postoperative chemotherapy for elderly CRC patients, they tend to receive postoperative chemotherapy less frequently than nonelderly patients. In our study, only 42.2% of patients with high-risk stage II disease and 46.5% of patients with stage III disease received postoperative therapy. Similarly, previous studies have reported that the proportion of elderly patients who received postoperative chemotherapy ranged from 15–57% [20–23]. These proportions are much lower than those in younger patients. Reasons for nontreatment and treatment interruptions in the elderly include comorbidities, side effects, and patient choice [23]. Although SIOG has recommended the performance of a comprehensive geriatric assessment (CGA) before cancer treatment [24], CGA is not frequently used in clinical practice in China. Considering that the comorbidities and tolerance of side effects by the elderly may affect the postoperative chemotherapy duration and outcome, we utilized clinical data from the electronic medical record system to calculate the CCI and then adjusted the CCI in Cox proportional hazard models.

Our study has some strengths. To our knowledge, this is the first study to evaluate the role of postoperative capecitabine duration in elderly patients with stage III and high-risk stage II colon cancer. This is the first report of a significant association between postoperative capecitabine duration and CSS in surgery-treated elderly colon cancer patients. Furthermore, an L-shaped relationship between capecitabine duration and CSS in stage III elderly colon cancer was discovered, and a threshold effect of capecitabine duration on CSS in this group was found. Strict statistical adjustment was applied to minimize residual confounders in this study.

There are still some limitations of our study. First, we could not evaluate the effect of postoperative chemotherapy duration of 5-fluorouracil alone or other regimens since the regimen applied for elderly patients with colon cancer was capecitabine in both medical centers. A multicenter study is necessary to extract more conclusions. In addition, the number of cases with the endpoint was small since only 43 (43/257) deaths occurred during our follow-up. It may be possible to increase the robustness of this study by extending the follow-up time to increase the number of end-point events. Second, bias could be caused by unmeasured confounders and residuals, but the sensitivity analysis indicated that the association between postoperative chemotherapy duration and CSS was robust. Third, dose reduction, significant delay, and compliance were not reported. Previous studies indicated decreased renal function and an increased number of comorbidities were correlated with reductions of the capecitabine dose [25]. Patients may need to receive > 70% relative dose intensity to improve their 5-year OS [26]. Currently, a trial is ongoing to explore this issue [27].

## Conclusions

In conclusion, the postoperative capecitabine duration was significantly associated with a decrease in death risk and recurrence risk in elderly stage III and high-risk stage II colon cancer. However, a threshold effect of capecitabine duration on survival was found with a turning point at 16 weeks. A 16-week regimen of capecitabine chemotherapy may improve the survival of elderly stage III colon cancer patients.

## Data Availability

The dataset supporting the conclusions of this article is included within the article.

## Abbreviations

CRC: Colorectal cancer
IDEA: International Duration Evaluation of Adjuvant Therapy collaboration
CAPOX: Oxaliplatin plus capecitabine
FOLFOX: Oxaliplatin plus 5-fluorouracil/leucovorin
RCTs: Randomized controlled trials
CSS: Cancer specific survival
CCI: Charlson comorbidity index
MMR: Mismatch repair
CA199: Carcinoma antigen 199
CEA: Carcinoembryonic antigen
HR: Hazard ratio
CI: Confidence interval
dMMR: Mismatch repair deficiency
SIOG: International Society of Geriatric Oncology
CGA: Comprehensive geriatric assessment
